# Cardiovascular Diseases and Type D Personality: Systematic Review and Meta-Analysis of the Literature of the Last 10 Years

**DOI:** 10.3390/life15071061

**Published:** 2025-07-02

**Authors:** Omar Anwar Saleh Al Nakhebi, Raluka Albu-Kalinovic, Adela Bosun, Oana Neda-Stepan, Marius Gliga, Cătălina-Angela Crișan, Ileana Marinescu, Cristian Mornoș, Virgil-Radu Enatescu

**Affiliations:** 1 Doctoral School, “Victor Babes” University of Medicine and Pharmacy, 300041 Timisoara, Romania; raluka.kalinovic@umft.ro (R.A.-K.); adela.bosun@umft.ro (A.B.); oana.neda-stepan@umft.ro (O.N.-S.); marius.gliga@umft.ro (M.G.); 2 Department of Neurosciences, Iuliu Hatieganu University of Medicine and Pharmacy, 400347 Cluj-Napoca, Romania; ccrisan@umfcluj.ro; 3 Faculty of Medicine, University of Medicine and Pharmacy of Craiova, 200349 Craiova, Romania; ileana.marinescu@umfcv.ro; 4 Department of Cardiology 1, “Victor Babes” University of Medicine and Pharmacy, 300041 Timisoara, Romania; cmornos@cardiologie.ro; 5 Department of Neurosciences, “Victor Babes” University of Medicine and Pharmacy, 300041 Timisoara, Romania; enatescu.virgil@umft.ro

**Keywords:** cardiovascular disease, type D personality

## Abstract

**Background**: Cardiovascular diseases (CVDs) are a leading cause of death globally, with a significant impact on public health and quality of life. In addition to traditional risk factors such as hypertension, diabetes, smoking, and hypercholesterolemia, a growing body of scientific evidence suggests the crucial role of psychosocial factors, including personality, in the development and prognosis of CVDs. In recent decades, researchers have focused on the so-called “type D personality.” **Methods**: Using the Preferred Reporting Items for Systematic Reviews and Meta-Analyses (PRISMA) guidelines, a literature search was conducted using the PubMed/Medline, Scopus, and Cochrane databases. Fifteen studies were included in the final quantitative analysis, involving 5687 people. The qualitative assessment of the studies included in this meta-analysis was conducted adopting the Quality System Tool. **Results**: Several studies have shown a significant correlation between type D personality and an increased risk of CVDs, as well as a worse prognosis in patients with established CVDs. The overall quality of the studies included in this synthesis ranged from 0.70 to 0.96, indicating the general good quality levels of the studies (69%). The association between type D personality and CVDs in terms of prevalence underlined a raw proportion of 0.36. **Conclusions**: This study reinforces the significance of type D personality as a psychological risk factor for CVD, with implications for both disease prevalence and severity.

## 1. Introduction

Cardiovascular diseases (CVDs) are reported as one of the most widespread causes of death worldwide, which, despite local and regional differences, are associated with 31.8% of deaths worldwide [1]. This high rate of mortality and related morbidity rate are due to multiple risk factors, and many educational programs are focused on preventing CVDs. As reported by Adhikary et al., these programs include strategies to improve healthy lifestyles, reduce smoking habits, increase physical activity, and learn adaptive nutritional styles in an attempt to change modifiable risk factors [2]. The most recent research on the etiopathogenesis and prognosis of CVDs is described from a biopsychosocial perspective that is not exclusively focused on the biological and physical dimensions of health but also includes psychological factors and relational and lifestyle characteristics [2]. This perspective is supported and followed by many organizations around the world that are focused on heart health. For example, the American Heart Association stated the importance of the relationship between psychological and cardiovascular health, suggesting the importance of screening the former in patients reporting heart issues [3].

Many empirical studies confirm that psychological well-being is associated with physical health due to the impact of mental processes on biological ones. In particular, the psychological factors that appear most associated with CVDs and that have been explored for a long time include stress, mood alterations such as depression and anxiety, and some peculiar personality configurations defined as type A and type D personalities [4,5]. In these latter cases, if a type A personality refers to individuals that are highly competitive and success-driven and that are characterized by hypervigilance and control and who may be affected by CVDs due to their levels of hypervigilance and tendency to act out negative emotion, thereby affecting their physical health, a type D personality, defined by Denollet in 1995, refers to the opposite pattern [5,6].

A type D personality is characterized by negative affectivity and social inhibition that does not allow for the reported high levels of distress to be expressed, and all the emotions (e.g., hostility, anger, irritation) manifest as somatization, consequently affecting heart health. Individuals with a type D personality are pessimistic, anxious and socially inhibited and have difficulty establishing relationships. Negative affectivity leads to a strong emotional reaction to stress, while social inhibition limits the search for support and the expression of emotions, causing isolation and discomfort. As reported by several studies, this personality is associated with a three-fold increased risk of adverse cardiovascular events such as myocardial infarction and cardiac death [7,8,9]. Also, in patients undergoing heart transplantation, type D personality has been associated with early rejection and increased mortality [10]. Some mechanisms underlying the association between type D personality and CVDs include high levels of inflammatory markers and a higher kynurenine/tryptophan ratio [11]. Inflammation and kynurenine/tryptophan metabolism mediate the effect of type D personality on plaque vulnerability and subsequent major adverse cardiac events. However, despite its early theorization and definition, type D personality and its characteristics have only more recently been frequently reported in patients with CVDs, and the reasons for this association are currently being explored, though it is still not clear if this personological framing represents a risk factor for specific heart failure or is a transdiagnostic dimension of CVDs. Moreover, heterogenous results have been reported, and there is a lack of clear data on the prevalence of heart pathologies in the presence of type D personality [12].

This meta-analysis was developed to summarize the evidence on the association between CVDs and type D personality. The possibility to elucidate the association between these dimensions from a biopsychosocial perspective may help in identifying an aspect that can reduce the high mortality and morbidity risk for CVDs reported by studies, working in a preventive form. From an applicative perspective, the findings of this work offer a critical synthesis of existing evidence, providing valuable insights for healthcare professionals. They highlight the importance of recognizing and integrating type D personality assessment into CVD prevention and clinical management strategies and intervention planning. The aim of the meta-analysis was to report evidence on the role of type D as risk factor for the severity of CVD from a longitudinal perspective, a dimension that should be considered not only to prevent but also to treat the risk for exacerbation or relapse.

## 2. Materials and Methods

### 2.1. Research Strategies and Eligibility Criteria

This systematic review and meta-analysis was conducted according to PRISMA (Preferred Reporting Items for Systematic Reviews and Meta-Analyses) guidelines to evaluate the association between type D personality and cardiovascular issues and to fulfill the objective of this study [13].

The protocol for this review was prospectively registered in the PROSPERO (International Prospective Register of Systematic Reviews) database under the registration number CRD420251067916, available online https://www.crd.york.ac.uk/PROSPERO/view/CRD420251067916 (accessed on 9 June 2025).

A systematic literature search was performed using the PubMed, Scopus, and Cochrane databases from January 2019 to March 2025. The systematic search was carried out using the terms “Coronary Artery Disease” and “Type D personality” combined with the Boolean operator “AND”.

### 2.2. Eligibility Criteria

The research question for this systematic review and meta-analysis was formulated using the PICO (Population, Intervention, Comparison, Outcome) framework to define the study’s inclusion and exclusion criteria precisely and systematically, ensuring coherence and relevance [14]. To be included in the systematic review, articles had to meet the following inclusion criteria: (a) Population: randomized controlled trials, cross-sectional studies, and comparative studies analyzing evidence on the association between type D personality and CVD in patients aged 18 years or older; (b) Intervention: the presence of a type D personality diagnosed using the DS14 questionnaire; (c) Comparison: not applicable; and (d) Outcomes: ascertaining the presence of CVD in patients with a type D personality. This will allow us to identify potential risk factors and, consequently, help reduce the high risk of mortality and morbidity associated with CVD in this population. In addition, we aim to evaluate the effectiveness of existing therapeutic interventions, study the prevalence and distribution of CVD in relation to type D personality, and monitor the incidence of these conditions.

Articles had to be written in English and published between January 2014 and March 2025.

The following exclusion criteria were applied: articles written in languages different to English and systematic or theoretical reviews, case reports, and procedural protocols were excluded from this selection. Systematic reviews and meta-analyses were read and consulted.

In the first selection phase, after removing duplicates, all studies were downloaded into Microsoft Excel. Selection by title and abstract screening was carried out by two independent reviewers (O.A.S.A.N and R.A.K.). In case of discrepancies, a third reviewer (V.R.E.) was consulted, and consensus was sought through discussion.

The second phase of the systematic process, which involved full-text screening and the final inclusion decision, was conducted jointly by the reviewers, and a quality assessment showed which studies were eligible for meta-analysis.

### 2.3. Data Selection Process and Data Extraction

From each article, the relevant data was extracted and included and is summarized in a table, with information about the study (authors, the year of publication, and the country of the study), population (the main descriptive information of the sample such as size, the percentage of males, and age), characteristics relevant to the aim of the study (clinical conditions, the specific evaluation of type D personality, and assessment tool).

### 2.4. Qualitative Assessment: Risk of Bias of the Included Studies

The qualitative assessment of the studies included in this meta-analysis was conducted using the Quality System Tool for the evaluation of quantitative studies, a checklist of 14 items exploring the quality of the rationale, methodologies, results, and outcome exposure of each study [15]. The procedure was used to assign a score from 0 to 2 (0 = no; 1 = partial; 2 = yes) based on the study’s alignment with each item on the checklist. A summary score was computed for each paper by calculating a total sum and dividing it by the total possible sum, reporting a ratio of the overall quality. In accordance with the guidelines of the tool, we adopted a cutoff of 0.75 as a score to include the papers in this work. To define the quality of the study, it was considered strong if the score was greater than 0.80 (H), good if it was 0.71–0.79 (G), adequate if it was 0.50–0.70 (A), and limited when the summary score was less than 0.50 (L) (Table 1).

### 2.5. Meta-Analysis Strategy

The first analysis was conducted to calculate the pooled prevalence of the type D personality in CVDs using proportion meta-analysis via a random-effects model. Raw data were considered, and tau^2^, the Q-test for heterogeneity (Cochrane 1954), and the I^2^ statistic were reported. Subsequently, an analysis was carried out using the log odds ratio as an outcome measure. A random-effects model was fitted to the data. The amount of heterogeneity (i.e., tau^2^) was estimated using the DerSimonian–Laird (DL) estimator, reporting the Q-test for heterogeneity and the I^2^ statistic. A random-effects meta-analysis was performed utilizing the DL estimator, which continues to be one of the most used methods in biomedical and clinical research because of its computational ease, intuitive framework, and closed-form outcome [16,17,18]. Its inclusion in all key meta-analytic software tools (e.g., RevMan (RevMan 5.3), STATA (Stata/SE 17.0), R (R version 4.2.1)) ensures accessibility and reproducibility for researchers and clinicians alike [19]. Since its emergence, the DL method has been widely utilized in research, featuring several significant systematic reviews and Cochrane evaluations [20,21]. In a comprehensive empirical assessment of 920 Cochrane meta-analyses, DL has emerged as the most commonly utilized variance estimator, and it continues to serve as a standard reference in comparative simulation research [22]. In contrast to other estimators like REML or Bayesian methods that require iterative fitting and are more resource-intensive, DL offers quick and consistent estimates, rendering it especially appropriate for reviews involving multiple outcomes, subgroup analyses, or sensitivity assessments. Its broad recognition, clarity, and uniform application across clinical areas improve comparability among studies and aid in incorporating new discoveries into the larger evidence framework. The DL estimator was selected as the main approach in this research for these reasons.

Studentized residuals and Cook’s distances were used to examine whether studies were outliers and/or influential in the context of the model. Studies with a studentized residual larger than the 100 × (1 − 0.05/(2 × k)) percentile of a standard normal distribution are considered potential outliers. The rank correlation test and the regression test, using the standard error of the observed outcomes as a predictor, are used to check for funnel plot asymmetry. Publication bias was tested with a funnel plot and fail-safe N calculation using the Rosenthal test.

## 3. Results

### 3.1. Selection of the Studies

**Figure 1:**
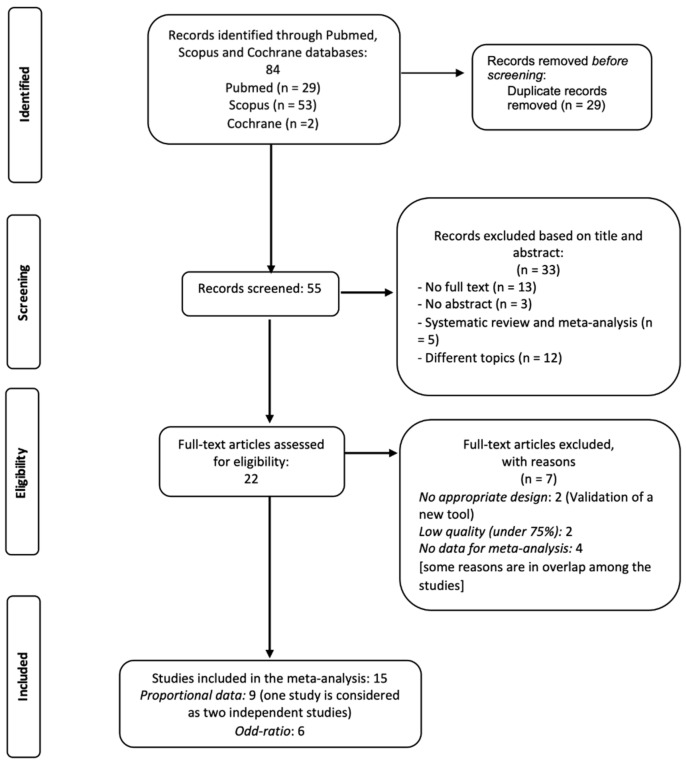
A summary of the search strategy employed in qualitative analysis.

The flowchart (Figure 1) shows the process of screening records. Initially, 84 records were identified. After removing duplicates (*n* = 29), the remaining 55 records were downloaded, screened based on titles and abstracts, and 33 records were excluded. The remaining 22 studies were screened by critically reading the full text. Finally, a total of 15 studies were included in the quantitative synthesis (Figure 1) [11,23,24,25,26,27,28,29,30,31,32,33,34,35,36].

### 3.2. Results of the Selected Studies

Table 2 presents the general characteristics of the included studies.

#### 3.2.1. Demographic Data

The 15 articles that met the inclusion criteria (we include 16 studies because Izquierdo Coronel et al. included two independent samples with MINOCA (myocardial infarction with non-obstructive coronary arteries) diseases (study 1) and MICAD (myocardial infarction with coronary arterial) diseases (study 2)) involved 5687 people. Participants were aged between 52 and 69.1 years, with the percentage of males ranging from 45% to 88.8%. One study did not report information about the participants’ gender. Six studies carried out a cross-sectional analysis, and nine studies performed a longitudinal evaluation with follow-ups ranging from 1 year to 10 years (Table 2). All the studies included in the quantitative synthesis adopted the same tool to evaluate type D personality (DS-14). One study carried out a cohort study with a follow-up assessment after 4.2 years [34]. Ten studies considered the prevalence of type D personality in a sample of clinical cardiovascular conditions (Table 2) with a mixed degree of severity and clinical characteristics. Six studies compared the presence of cardiovascular events in type D and non-type D personalities. Accordingly, a separate analysis was conducted.

#### 3.2.2. Qualitative Assessment

**Figure 2:**
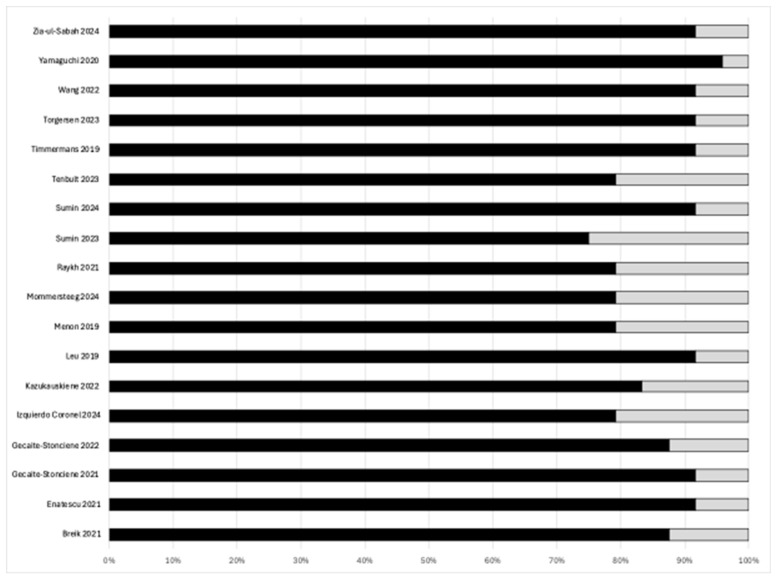
Quality reported by each study [11,23,24,25,26,27,28,29,30,31,32,33,34,35,36,37,38,39].

The overall quality of the studies included in this synthesis ranged from 0.70 to 0.96, reporting generally good levels of study quality (*n* = 11; 69%), but only one study reported high quality, with the highest scores in the checklist except for items 5, 6, and 7. Table 3 and Figure 2 show the quality reported by each study.

#### 3.2.3. Prevalence of Type D Personality in CVD

**Figure 3:**
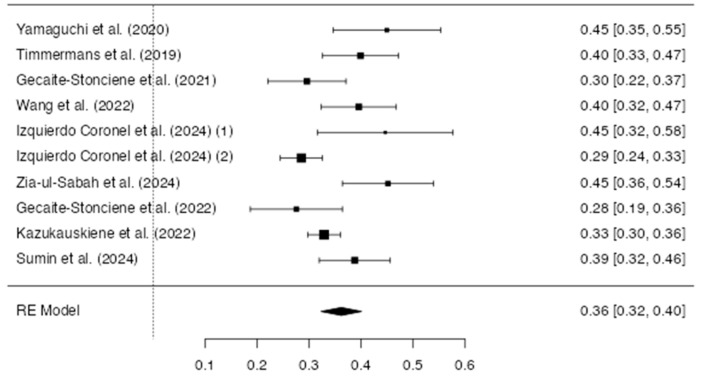
Forest plot of the studies reporting the prevalence of type D personality in CVD [11,25,26,27,32,33,35,36]. This forest plot summarizes the results of multiple studies. Each line represents a single study, showing its estimated effect and 95% confidence interval. The size of each square reflects the weight of the study in the overall analysis. The diamond at the bottom represents the combined effect across all studies.

**Figure 4:**
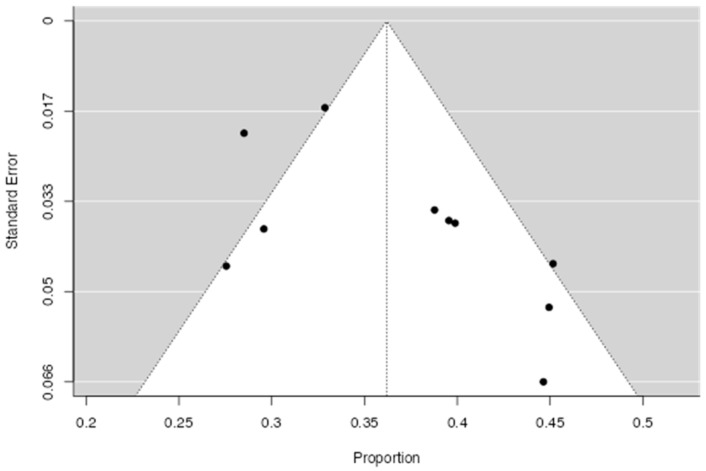
A funnel plot of the studies reporting the prevalence of type D personality in CVD.

The association between type D personality and CVDs in terms of prevalence underlined a raw proportion of 0.36 (95% CI [0.32–0.40]) (Figure 3). There was marked heterogeneity between studies (I^2^ = 70.7%). Additional analyses were conducted to examine the results for publication bias. The Rosenthal test for bias indicated significant asymmetry (Z = 2.14; *p* = 0.03). A visual exploration of the funnel plot showed moderate asymmetry (Figure 4).

#### 3.2.4. Difference Between Type D and Non-Type D in CVD

**Figure 5:**
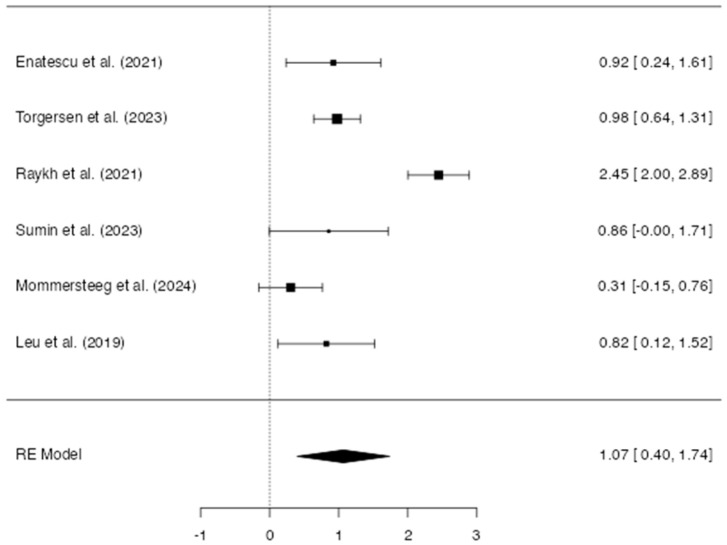
The forest plot of the studies indicates an odds ratio for CVD in type D personality [23,28,29,30,31,34]. This forest plot summarizes the results of multiple studies. Each line represents a single study, showing its estimated effect and 95% confidence interval. The size of each square reflects the weight of the study in the overall analysis. The diamond at the bottom represents the combined effect across all studies.

A total of k = six studies were included in the analysis. The observed log odds ratios ranged from 0.3051 to 2.4466, with most estimates being positive (100%). The estimated average log odds ratio was 1.08 (95% CI: 0.39 to 1.74) (Figure 5). Therefore, the average outcome differed significantly from zero (z = 3.12, *p* = 0.002). According to the Q-test, heterogeneity among the outcomes was observed (Q(5) = 48.56, *p* < 0.0001, tau^2^ = 0.61, I^2^ = 89.70%). A 95% prediction interval for the true outcomes ranged from –0.61 to 2.74. The 95% prediction interval observed in this analysis ranged from –0.61 to 2.74, encompassing both potential harm and substantial benefit. This wide interval signals a high level of between-study heterogeneity, indicating that the true effect in future settings may vary considerably from the pooled estimate. Unlike confidence intervals, which quantify uncertainty around the mean effect, prediction intervals account for the distribution of true effects across studies and provide an estimate of where the effect size of a new study is likely to fall [40,41]. Such a wide prediction interval (PI) has critical implications for clinical and policy decision-making. Even though the meta-analysis yielded a point estimate suggesting an average beneficial effect, the inclusion of negative values in the PI implies that, in certain contexts, the same intervention could be ineffective or even detrimental. This reflects real-world variability due to differences in population characteristics, intervention fidelity, study design, or outcome measurement across the included studies.

**Figure 6:**
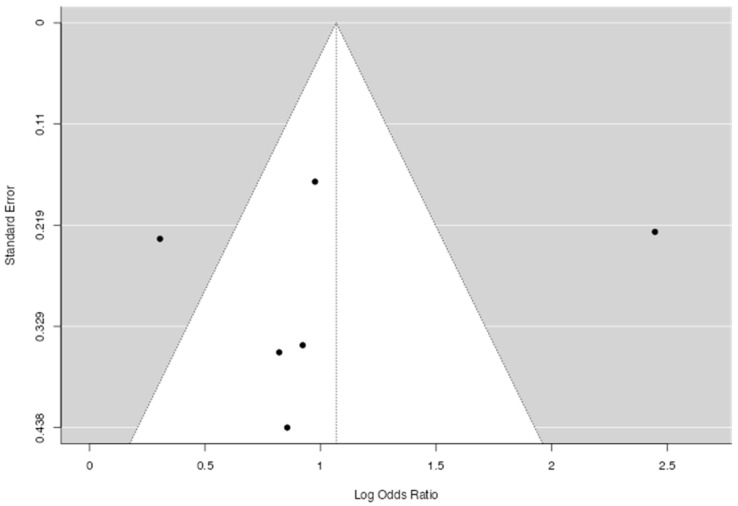
A funnel plot of the studies indicating the odds ratio for CVD in type D personality.

Hence, although the average outcome is estimated to be positive, in some studies, the true outcome may be negative. An examination of the residuals revealed that one study [30] had a value larger than ±2.64 and may be a potential outlier in the context of this model. According to Cook’s distances, none of the studies were overly influential. Neither the rank correlation nor the regression test indicated any funnel plot asymmetry (*p* = 1.00 and *p* = 0.67, respectively) (Figure 6).

## 4. Discussion

The findings of this meta-analysis provide robust evidence supporting a significant association between type D personality and an increased risk of CVDs. The pooled prevalence analysis reveals that approximately 36% of individuals with CVDs exhibit type D personality traits, highlighting a considerable proportion of patients potentially more vulnerable to adverse cardiovascular outcomes due to the impact of their psychological profile on stress management. This observation aligns with the previous literature, which emphasizes the role of psychological distress, social inhibition, and negative affectivity in exacerbating cardiovascular conditions [10,42]. Furthermore, a recent and extensive study by the Global Cardiovascular Risk Consortium has highlighted the relevance of focusing on psychological factors as potential risk factors for CVDs and heart failure, noting a persistent gap in the exploration of psychosocial risks despite the identification of numerous modifiable risk factors in cohort studies [43].

The odds ratio analysis further reinforces the notion that type D personality is not merely prevalent in people with CVDs but may actively contribute to the severity and progression of the disease, prognosis, and overall clinical presentation. The estimated average log odds ratio indicates that individuals with type D personality have a significantly higher likelihood of experiencing worse cardiovascular outcomes compared to their non–type D counterparts, considering the severity of the condition, the need for hospitalization, and the risk of recurrence [23,31,34].

It is important to note the presence of substantial heterogeneity among the included studies, suggesting that the characteristics of the samples, which differ in terms of the severity and nature of cardiac events and pathologies, as well as the study design and assessment timelines, may influence the results. Interestingly, despite the robust overall association, some studies have shown divergent trends. For instance, the study by T. Mommersteeg et al. did not report a predictive role of type D personality in the outcomes of major adverse cardiovascular events, suggesting the potential role of moderating factors such as demographic characteristics, cultural influences, or specific medical interventions [12,29].

It is important to note that while our analysis has focused on type D personality, the landscape of psychological factors in cardiovascular risk has also historically included type A personality. Characterized by ambition, competitiveness, impatience, and hostility, type A personality has been a subject of research since the 1950s. However, its association with cardiovascular disease has been more controversial and less consistent compared to what has been observed for type D. While some initial studies suggested a significant link, particularly with myocardial infarction, subsequent research has often shown a weaker or even absent relationship, with the exception of hostility emerging as the trait most consistently associated with elevated cardiovascular risk.

An interesting aspect contributing to this correlation is physiological activation. Individuals with type A personalities, especially those with high levels of hostility, tend to exhibit a more pronounced stress response. In situations perceived as highly stressful or challenging, these individuals may show an excessive activation of the hypothalamic–pituitary–adrenal (HPA) axis, leading to elevated and prolonged cortisol levels. Cortisol, known as the “stress hormone,” when in chronic excess, can have detrimental effects on the body, including systemic inflammation, endothelial dysfunction, insulin resistance, and hypertension. All these factors are well-known contributors to atherosclerosis and the development of cardiovascular disease. Therefore, the tendency of type A individuals to overreact to stressful situations with high cortisol secretion could represent one of the biological mechanisms linking this personality type to cardiovascular risk, especially when stress management is ineffective.

Type D personality, on the other hand, offers a more specific and replicable model of psychological vulnerability, focusing on emotional distress and social inhibition, which seem to act as more direct mechanisms in worsening cardiovascular outcomes [15,44,45].

### 4.1. Clinical Implications and the Role of Psychological Assessment

From a clinical perspective, the findings of this meta-analysis confirm the crucial importance of integrating psychological assessments into routine cardiovascular risk evaluations. Recognizing that a significant percentage of patients with CVDs exhibit type D personality traits underscores the need for a more holistic approach to risk management. The early identification of at-risk individuals through validated psychological screening tools can facilitate the implementation of targeted interventions. These interventions should focus on mitigating the negative impact of type D personality traits by promoting more adaptive coping strategies, reducing social inhibition, and modulating negative affectivity, with the ultimate goal of preventing cardiovascular risk and improving outcomes in patients with CVDs. Furthermore, it is essential for healthcare professionals to be aware of the potential reluctance of type D individuals to seek medical assistance or adhere to treatment regimens due to their tendency towards social isolation and emotional distress. An empathetic and personalized approach can improve treatment adherence and patient engagement.

### 4.2. Possible Biological Mechanisms

Several biological and behavioral mechanisms may contribute to the observed association between type D personality and CVD outcomes. At a biological level, the dysregulation of the immune system has been observed in individuals with type D personality, manifesting as elevated levels of pro-inflammatory mediators, such as C-reactive protein and cytokines [11], suggesting a link between chronic psychological stress and immune system dysregulation. The most studied cytokines in relation to type D personality and CVD are interleukin-6 (IL-6) and tumor necrosis factor-alpha (TNF-α). Elevated levels of IL-6 are associated with an increased risk of cardiovascular events, the progression of atherosclerosis, and poorer prognosis in patients with heart failure. TNF-α, another potent pro-inflammatory cytokine, contributes to endothelial dysfunction, the formation of atherosclerotic plaques, and adverse cardiac remodeling. Other relevant inflammatory mediators may include interleukin-1β (IL-1β) and C-reactive protein (CRP), a systemic marker of inflammation whose elevation is an independent predictor of future cardiovascular events. Low-grade chronic inflammation, promoted by the aforementioned mediators, is a known risk factor for atherosclerosis and other cardiovascular events, from initial endothelial dysfunction to plaque formation and rupture. Pro-inflammatory cytokines can induce the expression of adhesion molecules on endothelial cells, facilitating the infiltration of monocytes into the arterial wall. They also promote the proliferation of smooth muscle cells, lipid accumulation, and the production of reactive oxygen species, contributing to the instability of atherosclerotic plaques and increasing the risk of thrombotic events.

Furthermore, studies have confirmed a higher cortisol response in patients with CVDs and type D personality traits [24], indicating a dysregulation of the hypothalamic–pituitary–adrenal (HPA) axis. The HPA axis is the body’s primary stress response system. In conditions of chronic stress, such as that often experienced by individuals with type D personality, this axis can become dysregulated, leading to altered cortisol secretion and the sustained activation of the sympathetic nervous system, contributing to increased cardiovascular stress through various mechanisms, including increased blood pressure, heart rate, and vasoconstriction. At a behavioral level, individuals with type D may adopt fewer healthy lifestyles, such as a poor diet, a lack of physical activity, and smoking habits, and may have lower adherence to medical treatments, negatively impacting the progression of cardiovascular disease.

In summary, both the increase in inflammatory mediators and the dysregulation of the HPA axis represent plausible biological mechanisms through which type D personality can increase the risk and severity of cardiovascular diseases. Understanding these biological and behavioral mechanisms is of fundamental importance for the development of comprehensive and targeted interventions in the management of patients with type D personality and CVDs.

### 4.3. Types of Markers of Type D

The link between type D personality and an increased risk of cardiovascular disease has been extensively documented in research, supporting its inclusion in prevention guidelines. However, it is crucial to acknowledge that differences in the specific type D markers used across various sources can be a significant confounding factor and a limitation in the robustness of the findings.

While the type D Scale-14 (DS14), a 14-item instrument assessing negative affectivity and social inhibition, is recognized as the standard and most validated tool for identifying this personality type, not all studies rigorously adhere to its use or its specific cutoff criterion [46].

The use of alternative instruments that measure related psychological constructs, such as generic scales for anxiety, depression, or neuroticism, or variations in classification thresholds can lead to methodological heterogeneity. This heterogeneity not only complicates the comparability and synthesis of the results across studies (e.g., in meta-analyses) but also compromises the validity of the construct. This is because instruments not specifically designed for type D might not capture the unique interaction between negative affectivity and social inhibition that defines this personality [12,46].

Consequently, accurately assessing the clinical impact of type D personality on cardiovascular prognosis requires greater standardization in measurement tools and a critical awareness of the potential limitations arising from divergent methodological approaches.

### 4.4. Limitations and Future Directions

Despite the strengths of this meta-analysis, certain limitations must be acknowledged. First, the presence of publication bias, as suggested by funnel plot asymmetry, indicates a potential overestimation of the association between type D personality and CVDs. Studies with null or negative findings may be under-represented, leading to an inflated effect size. Additionally, the self-reported measures for assessing type D personality introduce the possibility of response biases, and future studies should consider incorporating more objective psychological assessments of the construct. Another important limitation is the heterogeneity observed across studies, which may stem from differences in sample characteristics, study designs, and measurement tools. Future research should explore potential moderators, such as sex differences, socioeconomic factors, and comorbid psychological disorders, to refine our understanding of the precise role of type D personality in cardiovascular health, as suggested by the global cardiovascular risk consortium [11]. Longitudinal studies with extended follow-up periods over the reported years of the analyzed studies on the association between type D personality and CVD progression are welcome to understand how type D traits influence cardiovascular trajectories over time in aging.

## 5. Conclusions

In summary, this meta-analysis reinforces the significance of type D personality as a psychological risk factor for CVDs, with implications for both disease prevalence and severity. These findings highlight the necessity of integrating psychological screening into cardiovascular care and developing tailored interventions to address the unique challenges faced by type D individuals. Further research should aim to refine risk stratification models, explore intervention efficacy, and elucidate the underlying biological mechanisms linking psychological traits to cardiovascular outcomes.

## Figures and Tables

**Table 1:** Checklist of Quality System Tool.

Checklist
Question/objective sufficiently described?
2. Study design evident and appropriate?
3. Method of subject/comparison group selection or source of information/input variables described and appropriate?
4. Subject (and comparison group, if applicable) characteristics sufficiently described?
5. If interventional and random allocation was possible, was it described?
6. If interventional and blinding of investigators was possible, was it reported?
7. If interventional and blinding of people was possible, was it reported?
8. Outcome and (if applicable) exposure measure(s) well defined and robust to measurement/misclassification bias? Means of assessment reported?
9. Sample size appropriate?
10. Analytic methods described/ justified and appropriate?
11. Some estimate of variance was reported for the main results?
12. Controlled for confounding?
13. Results reported in sufficient detail?
14. Conclusions supported by the results?

**Table 2:** General characteristics of the included studies.

S. No	First Author’s Name/Year of Publication [Reference Number]	Country	Sample Size	Male Percentage	Age (Mean; Age Range)	Study Design	MA Data	Clinical Condition	Outcomes
1	Enatescu/2021 [23]	Romania	221	59.3	60 ± 10.2	Cross-sectional	Odds Ratio	CAD (coronary artery disease)	Type D is associated with higher CVD severity (IMA).
2	Gecaite-Stonciene/2021 [24]	Lithuania	142	85	52 ± 8	Cross-sectional	Prevalence	ACS (Acute Coronary Syndrome)	Type D is associated with higher distress and lower cardiovascular response.
3	Gecaite-Stonciene/2022 [25]	Lithuania	98	87.8	52.92 ± 7.17	Cross-sectional	Prevalence	CAD	Type D was associated with CAD (cortisol response).
4	Izquierdo Coronel/2024 (1) [26]	Spain	56	45	66.8 ± 13.7	Longitudinal study (FU: 3 years)	Prevalence	MINOCA (myocardial infarction with non-obstructive coronary arteries)	Type D is associated with a cardiovascular condition.
5	Izquierdo Coronel/2024 (2) [26]	Spain	477	76	66.5 ± 13.7	Longitudinal study (FU: 3 years)	Prevalence	MICAD (myocardial infarction with coronary artery disease)	Type D is associated with a cardiovascular condition.
6	Kazukauskien/2022 [27]	Lithuania	864	74	58 ± 9	Longitudinal study (FU: 5 years)	Prevalence	CAD	Type D personality is associated with a lower quality of life.
7	Leu/2019 [28]	Taiwan	777	84.3	62.03 ± 10.5	Longitudinal study (FU: 1 year)	Odds Ratio	CAD	Type D personality predicts CAD.
8	Mommersteeg/2024 [29]	The Netherlands	517	55	63	Longitudinal study (FU: 10 years)	Odds Ratio	MACE (major adverse cardiovascular event)	Type D does not predict negative outcomes in MACE.
9	Raykh/2021 [30]	Russia	602	81.4	57.7 ± 7.3	Longitudinal study (FU: 10 years)	Odds Ratio	Post-coronary artery bypass grafting	Negative outcomes of CAD at 5 years post intervention are highly associated with Type D.
10	Sumin/2023 [31]	Russia	91	-	64.7	Longitudinal study (FU: 1 year)	Odds Ratio	MACE	Type D personality predicts MACE events and hospitalization.
11	Sumin/2024 [32]	Russia	196	73	62 ± 1	Longitudinal study (FU: 10 years)	Prevalence	CAD	Type D is predictive of the prognosis of CAD.
12	Timmermans/2019 [33]	The Netherlands	173	77	69.1 ± 9.6	Cross-sectional	Prevalence	CAD	Type D personality is associated with a low quality of life (physical health status) and social anxiety.
13	Torgersen/2023 [34]	Norway	1083	79	61.5 ± 9.6	Cohort study; FU: 4.2 years	Odds Ratio	CAD	Type D personality is associated with recurrent CAD.
14	Wang/2022 [11]	China	177	58.8	55.79 ± 10.70	Cross-sectional	Prevalence	CAD	Type D is associated with poor cardiovascular outcomes in CAD, mediated by a pro-inflammatory biomarker.
15	Yamaguchi/2020 [35]	Japan	89	88.8	66 (58–74)	Cross-sectional	Prevalence	CAD	Type D personality in CAD is associated with depression and negative coping.
16	Zia-ul-Sabah/2024 [36]	Saudi Arabia	124	55	67 ± 10	Longitudinal (FU: 1 year)	Prevalence	Post-STEMI	Type D is predictive of the severity of left ventricular adverse remodeling.

**Table 3:** Risk of bias evaluation of the included Quality System Tool.

S. No	First Author’s Name/Year of Publication [Reference Number]	Item_1	Item_2	Item_3	Item_4	Item_5	Item_6	Item_7	Item_8	Item_9	Item_10	Item_11	Item_12	Item_13	Item_14	Total Score	Index of Quality	Risk of Bias	Index of Quality	
1	Enatescu/2021 [23]	2	2	2	2	0	1	0	1	2	2	2	2	2	2	22	0.92	G	92	8
2	Gecaite-Stonciene/2021 [24]	2	2	2	2	0	0	0	2	2	2	2	2	2	2	22	0.92	G	92	8
3	Gecaite-Stonciene/2022 [25]	2	2	2	2	0	0	0	2	2	1	2	2	2	2	21	0.88	G	88	13
4	Izquierdo Coronel/2024 [26]	2	1	1	1	0	0	0	2	2	2	2	2	2	2	19	0.79	A	79	21
5	Kazukauskien/2022 [27]	2	1	2	1	0	0	0	2	2	2	2	2	2	2	20	0.83	G	83	17
6	Leu/2019 [28]	2	2	2	2	0	0	0	2	2	2	2	2	2	2	22	0.92	G	92	8
7	Mommersteeg/2024 [29]	2	1	2	1	0	0	0	2	1	2	2	2	2	2	19	0.79	A	79	21
8	Raykh/2021 [30]	2	1	2	1	0	0	0	1	2	2	2	2	2	2	19	0.79	A	79	21
9	Sumin/2023 [31]	2	1	1	1	0	0	0	2	1	2	2	2	2	2	18	0.75	A	75	25
10	Sumin/2024 [32]	2	2	2	2	0	0	0	2	2	2	2	2	2	2	22	0.92	G	92	8
11	Timmermans/2019 [33]	2	2	2	2	0	0	0	2	2	2	2	2	2	2	22	0.92	G	92	8
12	Torgersen/2023 [34]	2	2	2	2	0	0	0	2	2	2	2	2	2	2	22	0.92	G	92	8
13	Wang/2022 [11]	2	2	2	2	0	0	0	2	2	2	2	2	2	2	22	0.92	G	92	8
14	Yamaguchi/2020 [35]	2	2	2	2	1	0	0	2	2	2	2	2	2	2	23	0.96	H	96	4
15	Zia-ul-Sabah/2024 [36]	2	2	2	2	0	0	0	2	2	2	2	2	2	2	22	0.92	G	92	8
16	Breik/2021 [37]	2	2	2	1	0	0	0	2	2	2	2	2	2	2	21	0.88	G	88	13
17	Menon/2019 [38]	2	1	1	2	0	0	0	2	1	2	2	2	2	2	19	0.79	A	79	21
18	Tenbult/2023 [39]	2	1	1	1	0	0	0	2	2	2	2	2	2	2	19	0.79	A	79	21

## Data Availability

All research data are present in the manuscript.
